# The impact of population aging on income gaps: Can digital finance play a buffering role?

**DOI:** 10.1371/journal.pone.0340310

**Published:** 2026-04-01

**Authors:** Zhongyuan Sun, Ying Dong, Guangfeng Ning

**Affiliations:** 1 School of Economics and Management, Xi’an University of Posts and Telecommunications, Xi’an, China; 2 Business School, Fuyang Normal University, Fuyang, China; 3 School of Economics and Management, Beijing University of Posts and Telecommunications, Beijing, China; Pusan National University College of Economics and International Trade, KOREA, REPUBLIC OF

## Abstract

Drawing on the panel data of 284 prefecture-level cities in China spanning from 2011 to 2022, this study empirically examines the impact and underlying mechanisms of population aging on urban income disparities through regression analysis. Additionally, it delves into the moderating role of digital finance in this relationship. The main conclusions are as follows: firstly, population aging has asignificant positive impact on both intra-urban income gap and inter-urban income gap. The heterogeneity analysis indicate that the impact of population aging on the inter-urban income gap cities is more significant in the samples of underdeveloped cities and resource-based cities. Secondly, population aging reduces the labor income share and increases social security expenditure, thus expanding the intra-urban income gap. Meanwhile, population aging enlarges the inter-urban income gap by reducing the technological innovation and impeding industrial structure upgrading. Finally, digital finance effectively alleviates the impact of population aging on the intra-urban income gap and inter-urban income gap. Further research reveals that as the quantile of the intra-urban income gap rises, the marginal impact effect of population aging on intra-urban income gap generally demonstrates a trend of first rising and then declining. Meanwhile, as the quantile of the inter-urban income gap increases, the impact coefficient of population aging on the inter-urban gap tends to decline monotonically.

## Introduction

With the steady development of the global economy and remarkable advances in medical and health care, population aging has emerged as an irreversible and pivotal social trend across the globe. Taking China as a case in point, data from the “2024 Elderly Care Development Report” issued by the Ministry of Civil Affairs shows that, as of the end of 2024, the elderly population aged 65 and above in China had reached 220 million, representing 15.6% of the total population. Projections indicate that this proportion will surpass 20% by 2030. The accelerated advancement of population aging has posed severe challenges and new propositions for key domains including national social governance, the development of the elderly care service system, and economic structural transformation. Although population aging has spawned a huge “silver economy” market, beneath its superficial prosperity lies deep-seated structural contradictions, including a shrinking total labor supply and increasing operational pressure on the social security system. Against this realistic backdrop, the core paradox inherent in the “silver economy”—the dual superposition of market dividends and development concerns—is becoming increasingly prominent, emerging as a key issue that needs to be urgently addressed in current economic and social development. The paradox of the “silver economy” reveals the complexity and uncertainty of the impact of population aging on economic development. On the one hand, the large elderly population has given rise to new consumption demands, which hold the potential to become new drivers of economic growth [1]. On the other hand, due to the general decline in income and limited payment ability of the elderly population, as well as the potential crowding out of productive investment, their actual economic driving effect may be far lower than expected, and may even exacerbate social distribution pressure [2]. This inherent duality renders the implications of population aging for income distribution far more complex. At the intra-urban level, population aging may reinforce the Matthew Effect through channels such as pension disparities and unequal asset accumulation, widening the income gap both among the elderly population and across generations [3]. However, intra-household transfer payments and inclusive elderly care policies may also exert a redistribution effect. At the inter-urban level, the “Siphon Effect” of developed regions is likely to leverage the “silver economy” to gather resources, continuously attract the inflow of young labor force [4], leaving underdeveloped regions confronted with the dual challenges of labor shortage and intensified aging, thereby further widening the regional development gap. Meanwhile, factors such as industrial transfer and policy support may also help underdeveloped regions activate their local elderly care industries and achieve catch-up. The formation logics of intra-urban and inter-urban income gaps are fundamentally distinct. Examining the impact of population aging on income gaps from the dual perspectives of the intra-urban and inter-urban levels is not a mere superposition of dimensions, but rather a exploration of the effect of population aging on both intra-urban and inter-urban income gaps based on their inherent differences in driving mechanisms, social consequences, and policy implications. This approach is crucial to accurately grasping the essence of income polarization and providing a solid basis for differentiated regulatory policies. Furthermore, the complexity revealed by the “silver economy” paradox suggests that the impact of population aging on intra-urban and inter-urban income gaps is far from a simple linear relationship of increase or decrease. In-depth analysis and differentiation of the complex and dynamic interaction patterns between aging and income gaps are of invaluable foundational significance for constructing differentiated governance frameworks adapted to different regions and development stages.

Faced with the challenges posed by population aging to the income distribution structure, there is an urgent need to explore effective response measures. In recent years, backed by cutting-edge technologies such as big data, artificial intelligence, and blockchain, digital finance has boomed globally, demonstrating enormous potential to reshape the traditional financial landscape and promote economic and social development [5,6]. This provides new perspectives and potential tools for addressing the dilemma of income distribution against the backdrop of population aging. However, digital finance is essentially a techno-institutional complex characterized by high permeability and restructuring capacity. Whether it will ultimately act as a “stabilizer” for the widening of income gaps or unexpectedly function as an “accelerator” constitutes an urgent theoretical and empirical puzzle that demands in-depth investigation. From the perspective of a “stabilizer”, the inclusive nature of digital finance can indeed directly mitigate the financial exclusion triggered by population aging. At the intra-urban level, through mobile payments, digital credit, and robo-advisory services, digital finance empowers low-income elderly groups and their families, enhancing their financial accessibility and asset appreciation capacity [7], thereby restraining the Matthew Effect within cities arising from disparities in elderly care resources. At the inter-urban level, its geographical penetrability facilitates the reverse flow of capital, information, and services to underdeveloped regions, activating local economies and retaining human resources [8,9], mitigating the trend toward regional divergence dominated by the Siphon Effect and promoting coordinated regional development. However, the risks associated with its potential role as an “accelerator” should not be underestimated. The development and adoption of digital finance are not homogeneous, and its effectiveness is highly dependent on digital literacy, infrastructure, and supporting institutions. At the intra-urban level, disparities in digital access and usage capabilities may widen the “digital divide”, enabling groups with skills and resources to accumulate digital dividends more rapidly [10], thereby further expanding the wealth gap with vulnerable elderly groups. At the inter-urban level, the digital financial resources—along with data, technology, and talent—may further concentrate in developed cities, which in turn reinforces their status as financial hubs, intensifies the Siphon Effect on surrounding regions, further solidifies the “core-periphery” structure, and ultimately accelerates the widening of inter-urban income gap [11]. At the juncture of deepening population aging and the rapid development of digital finance in China, it is necessary to empirically verify whether digital finance primarily plays a regulatory and stabilizing role or has unintentionally exacerbated inequality. Clarifying this key issue is of vital practical significance for formulating targeted digital finance policies that are both inclusive and efficient for an aging society.

Building on the aforementioned complex impacts of population aging on income gaps and the emergence of digital finance as a potential mitigating tool, this study aims to explore the following core research questions. Firstly, how exactly does population aging affect intra-urban income gap and inter-urban income gap? Does a non-linear impact effect exist? Secondly, what are the internal mechanisms through which population aging exerts an influence on the intra-urban and inter-urban income gaps? Thirdly, can the developing digital finance effectively moderate the widening effect of population aging on the above two types of income gaps? To answer these questions scientifically, this study uses panel data from 284 prefecture-level cities in China spanning 2011–2022 and adopts the fixed-effects regression analysis method to systematically and empirically examine the dual impacts of population aging on urban income gaps and their operational mechanisms. It also focuses on analyzing the key moderating role played by digital finance in this process. The research contributions are mainly manifested in the following aspects. Firstly, this study simultaneously investigates the dual impacts of population aging on intra-urban income gap and inter-urban income gap, breaking through the limitation that existing literature predominantly focuses on a single dimension. Furthermore, it conducts an in-depth analysis of their non-linear impact effects. This comprehensive analysis more comprehensively reveals the complex influences of aging on the overall income distribution structure, and helps to understand how its spatial differentiation effect exacerbates regional development imbalance. Secondly, this paper not only verifies the direction of the impact of aging, but also deeply reveals the core mechanisms behind it. The empirical results show that at the intra-urban level, aging exacerbates intra-urban income inequality mainly through two key paths: reducing the labor income share and significantly increasing local governments’ social security expenditures. At the inter-urban level, aging widens the income gap between residents of different cities mainly through two mechanisms: inhibiting urban technological innovation and hindering industrial structure upgrading. The identification of these mechanisms provides a micro-foundation for understanding how aging reshapes the economic structure. Finally, this paper incorporates digital finance into the analytical framework, and systematically empirically tests its significant moderating role in the relationship between population aging and income gap. The study finds that digital finance can effectively weaken the widening effect of aging on both intra-urban and inter-urban income gaps. This provides direct and robust empirical evidence for leveraging the dividends of digital technology to address the challenges of aging.

The subsequent arrangements of this article are as follows: The second part is the literature review; the third part is the hypothesis development; the fourth part is the research methodology; the fifth part is the empirical results and discussion; the sixth part is the conclusions and policy implications.

## Literature review

### Population aging and income gap

Looking back at the 1950s and 1960s, numerous developed countries successively entered an aging society. As the proportion of the elderly population continued to increase, profound transformations took place in aspects such as the supply-demand relationship in the labor market and the burden on the social security system. Whether population aging will reshape the income distribution pattern and how it will do so have emerged as focal points in academic research. Paglin [12] was the first to put forward the concept of the “age effect”, emphasizing that the population age structure could be a crucial factor influencing individual income disparities, this concept has drawn extensive attention within the academic community. Scholars to date have vigorously delved into the influence of population aging on income inequality. However, the findings reached are far from uniform. A segment of studies are of the opinion that population aging is likely to exacerbate the income gap among residents. Based on the permanent income hypothesis, Deaton and Paxson [13] conducted an empirical analysis by using the household survey data from the United States, the United Kingdom, and Taiwan, China. The results indicate that the consumption inequality and income inequality indeed increase with age. Ohtake and Saito [14] carried out an empirical analysis of Japanese data and found that as the elderly population grows, the disparities in human capital levels and physical capital accumulation gradually become more prominent, ultimately exacerbating overall income inequality. Lin et al. [15] utilized the sample data from 22 regions in Taiwan, China, and discovered a positive correlation between population aging and the income gap. Kang and Rudolf [16] made use of the data from the Korean Household Income and Expenditure Survey and found that there is a remarkable inter-generational trend in the income gap, and the aging population has significantly widened the overall income gap. Dong et al. [17], Chen et al. [18], Liu and Sun [19] by utilizing the data from the China General Social Survey, also discovered that population aging would expand income inequality.

However, some scholars argue that population aging has led to a reduction in the income gap. For example, Morley [20], by analyzing Brazilian data, discovered that the younger population age structure brought about by immigration has widened income inequality. Mason and Lee [21] analyzed the data of Taiwan, China from 1978 to 1998 and found that population aging alleviated the income gap. Moreover, some scholars hold the view that the relationship between population aging and the income gap is rather intricate and non-linear. For example, Don [22] held that with the process of population aging, the income gap presents an inverted “N”-shaped characteristic. As population aging deepens initially, the income gap contracts. Once it reaches the trough, the income gap starts to expand. Subsequently, after hitting the peak, the income gap contracts once more. Wang et al. [23] believed that there is a “U”-shaped relationship between population aging and income inequality, before reaching a certain critical value, population aging has an inhibitory effect on income inequality, however, when this critical value is exceeded, it will widen income inequality. At the same time, some scholars have pointed out that the explanatory power of population aging for the income gap is so small that it can almost be ignored. For instance, Barreti et al. [24] using the Australian Bureau of Statistics Household Expenditure Surveys from 1975 to 1993, pointed out that both income inequality and consumption inequality increased significantly during this period, while the changes in population aging and family structure accounted for only a small part of the overall growth in economic inequality. Guo et al. [25] pointed out that in urban areas of China, the degree of income gap within the younger generation is more significant than that within the older generation. Population aging will bring about an income gap, but this impact gradually diminishes over time.

The impact of population aging on income gap is a complex and controversial topic. Existing research generally suggests that population aging may affect income distribution through various channels, but its specific effects vary depending on regional, economic structure, and social security systems. This article further explores the multidimensional impact of aging on income gap by dividing it into intra-urban income gap and inter-urban income gap. This research endeavors to unearth the hitherto overlooked inherent correlations between population aging and income inequality across diverse levels by means of meticulously refined analysis dimensions. The findings will serve as a solid theoretical underpinning for the formulation of highly targeted policies, thus contributing to more effective socioeconomic strategies in the context of an aging society.

### Digital finance and income gap

Digital finance refers to a new generation of financial services that integrate traditional financial service models with the help of Internet and information technology [26]. Digital finance provides financial services in a digitalized manner, bringing about numerous transformations to economic activities [27]. It breaks through the constraints of time and space, reduces the costs of financial services, and offers more people the opportunity to participate in economic activities [28]. Financial resources inherently possess the characteristics of profit-seeking and agglomeration. However, digital finance has broken through these limitations, significantly enhancing its permeability, usability, and affordability [29]. The improvement in the degree of permeability and usability will directly reduce financial costs. The human capital effect serves as a catalyst, propelling vulnerable groups to make the transfer towards more advanced production sectors, industries, and regions. This, in turn, effectively eases the income inequality that exists among sectors, industries, and regions, thereby fostering a more equitable economic landscape [30].

Regarding the research on the impact of digital finance on the income gap, some scholars believe that digital finance can reduce the income gap. Bittencourt [31] conducted research using Brazilian data and found that enhancing the level and scope of financial services in low-income areas is conducive to improving the living conditions of the 20% of the low-income population. Digital finance can significantly increase the income level of low-income groups through various mechanisms. Regarding the specific mechanisms, some scholars maintain that digital finance can break the limitations of physical branches in traditional financial services. Leveraging the Internet and mobile terminals, it extends financial services to remote regions and disadvantaged groups, thereby enabling wider access to financial resources that were previously out of reach [32]. By virtue of the threshold-reducing effect, digital finance surmounts barriers such as exorbitant handling fees and convoluted procedures. This empowers low-income individuals to readily access fundamental financial services, including savings, payment services, and credit facilities, which were once difficult for them to obtain due to the high-cost and complex-process nature of traditional financial services [33].

This effectively alleviates the financial exclusion phenomenon. It seamlessly integrates disadvantaged groups into the financial system, furnishing them with essential financial support for endeavors like entrepreneurship and investment. Consequently, it narrows the gap between low-income and high-income groups in terms of the accessibility and utilization efficiency of financial services, thus reducing the income gap [34,35]. At the same time, some scholars also believe that digital finance can optimize capital allocation, improve the efficiency of financial markets, and promote innovation, entrepreneurship, and the development of small and medium-sized enterprises [36,37]. On the one hand, it provides financing convenience for small and medium-sized enterprises, which will stimulate market vitality [38]. Thus, it creates more employment opportunities for low-income groups. With more jobs available, low-income earners can gain financial independence, enhance their skills through on-the-job training, and gradually move up the economic ladder [39]. On the other hand, digital finance spawns new industries and business models within the digital economy, like e-commerce platforms and mobile payment services. These emerging sectors offer low-threshold job opportunities for low-skilled workers. By enabling them to secure employment, their income levels are elevated. As a result, the income gap between different income groups is narrowed [40,41].

Conversely, certain studies indicate that digital finance has the potential to exacerbate the income gap. Yao and Ma [42] conducted a study using data from 280 prefecture-level cities in China, and found that the development of digital finance has a Kuznets effect on the income distribution of Chinese residents. Most regions in China have not yet crossed the inflection point of the bell-shaped curve. As digital finance develops, the income gap within these regions is expected to keep widening. At the same time, some scholars have also indicated that the existence of the digital divide means that some elderly people and low-income groups may not be able to fully utilize digital financial resources due to a lack of relevant knowledge and skills. This further exacerbates the income gap between them and those proficient in digital technologies [43]. In addition, it is relatively difficult to prevent and control the risks of digital finance, and there is a lag in supervision. Issues such as network security and information leakage may lead to property losses for users, and low-income groups with weaker risk-bearing capacity are more likely to be affected. Some scholars also believe that the impact of digital finance on the income gap is influenced by real-world conditions. For example, Altunbas and Thornton [44], through an analysis of 121 countries worldwide, found that the impact of financial development on income inequality varies with a country’s income level. It can promote income equality in upper-middle income countries, while in low-income and high-income countries, digital finance may instead exacerbate income inequality. Law et al. [45] argued that financial development is likely to reduce income inequality only when a certain threshold level of institutional quality is attained. Prior to reaching this threshold, the impact of financial development on income inequality is not significant.

Currently, research into the interactive relationships among population aging, income gap, and digital finance still has some deficiencies. Thoroughly exploring this field can further enhance the theoretical framework at the intersection of multiple disciplines like population economics, finance, and sociology. Through empirical analysis and model construction, quantifying the specific impact of digital finance on the income gap in various scenarios helps to deepen our understanding of the internal-urban connection between financial innovation and the transformation of the social-economic structure. As a result, it provides more copious theoretical support and research ideas for subsequent scholars’ research, enabling them to conduct more in-depth and comprehensive studies in this area. A profound exploration of the relationship between population aging and urban income gaps, along with an in-depth examination of how to fully capitalize on the advantages of digital finance, bears substantial practical significance for promoting social equity and propelling sustainable economic development.

## Hypothesis development

With the rising proportion of the elderly population, significant changes have occurred in multiple aspects such as labor markets, wealth distribution, and family structures, thereby exerting a significant impact on income distribution. Firstly, at the level of labor market, population aging reduces the overall labor force participation rate. The labor supply shortage caused by aging forces the transformation of economic growth from a factor-driven model to a technology-innovation-driven one [46,47], which in turn exacerbates the depreciation of human capital among some low-skilled elderly groups. Meanwhile,the characteristic of technological progress being biased towards capital and skills, coupled with the relatively weak adaptability of elderly workers, will further widen the wage income gap between high-skilled young workers and low-skilled elderly workers. Secondly, at the level of wealth distribution, population aging will intensify intergenerational wealth transfers, thereby expanding the income gap between families with different initial wealth accumulations [48]. China’s one-child policy has led to smaller and more homogeneous family structures. In this context, with the advancement of population aging, intergenerational transfers of family wealth have become more concentrated and pronounced. The younger generations of older, high-income groups with substantial stock assets can gain initial capital advantages through inheritance and support from family resources [49]. In contrast, the younger generations of low-income families, due to a lack of wealth inheritance, face dilemmas such as inadequate investment in human capital and constrained career development, which leads to the solidification and continuous expansion of income gap between generations. Finally, at the level of family structure, the population aging accompanied by low birth rates has increased the burden of elderly care for core families [50]. High-income households are more capable of alleviating this pressure by purchasing market-oriented services, maintaining their advantage in income accumulation. In contrast, low-income households, due to caregiving pressure, may be forced to reduce working hours or withdraw from the labor market. Over time, the gaps between different income groups within the city in terms of the speed of wealth accumulation, ability to access resources, and space for social mobility become in creasingly entrenched. Eventually, the Matthew effect in income distribution becomes more pronounced, further tearing apart the internal income stratification of the city.

Based on this, this paper puts forward the following assumptions:

**H1.** The population aging will expand the intra-urban income gap.

The acceleration of population aging will systematically widen the inter-urban income gap. The core lies in the fact that aging exerts differentiated impacts on cities with different endowments, and amplifies development gaps through labor supply, fiscal resource allocation and factor mobility. Firstly, at the level of labor supply, cities with deep and rapidly growing aging face a more severe shrinkage of the working-age population and an aging skill structure, thereby undermining their industrial competitiveness and innovation vitality [51]. In contrast, core metropolitan areas or cities with an inflow of young migrants can partially offset the negative impacts of aging by continuously attracting high-quality labor, and even strengthen their advantages in the knowledge-based economy. This leads to a further agglomeration of high-value-added industries and talents towards the economically developed cities [52], thereby expanding labor productivity and income levels in different regions. Secondly, from the perspective of fiscal resource allocation, local governments are forced to allocate more public expenditures to elderly security and medical care, crowding out investment in the cultivation of emerging industries and the upgrading of infrastructure, and weakening the competitiveness of the business environment [53]. A weakened business environment directly reduces a region’s attractiveness to high-quality enterprises and high-end factors, leading to the transfer of technology-intensive, high-value-added industries to regions with a better business environment. As a result, the local industrial structure becomes locked in low-value-added sectors, with stagnant growth in employment quality and income levels. In contrast, regions with a superior business environment continue to gain favor from capital and technology, forming a positive cycle of industrial upgrading and income growth. This ultimately leads to a “Matthew effect” across regions, further widening the income gap. Finally, at the level of cross-regional factor mobility, the degree of aging has become a key factor driving the selective migration of labor, capital, and even consumption capacity. The young labor force tends to flow into expanding cities characterized by a low degree of aging and less pressure on public services. High-quality capital, driven by the demand for industrial upgrading, agglomerates in regions rich in human capital, consumption capacity also concentrates in vibrant cities along with the migration of high-income groups [54]. This causes shrinking cities to fall into stagnation in income growth due to factor loss, while expanding cities accelerate income growth through factor agglomeration, further widening the inter-urban income gap.

Based on this, this paper puts forward the following assumptions:

**H2.** The population aging will expand the inter-urban income gap.

The process of population aging can significantly reduce the labor income share in national income distribution and widen the intra-urban income gap through the high inequality in the distribution of capital gains. Firstly, population aging leads to the accelerated substitution of capital for labor. Faced with shrinking labor supply—particularly the scarcity of young labor and the declining skill adaptability of elderly workers—enterprises tend to increase capital inputs such as automated equipment and artificial intelligence to maintain production. This results in a rise in the marginal contribution of capital factors within the production function, while that of labor factors relatively declines [55,56]. Secondly, capital-biased technological progress is reinforced. In response to rising labor costs and an aging population structure, technological innovation becomes more focused on labor-saving, which further squeezes the bargaining power of low-skilled workers and expands the income advantages of capital owners and technical elites [57]. Finally, there is a structural imbalance in labor-capital bargaining power. AAging is accompanied by a decline in union coverage, an increase in flexible employment, and a weakening of elderly workers’ ability to switch jobs, which in turn reduces the overall bargaining power of labor [58]. Meanwhile, the increased mobility of capital and the deepening of digital transformation have enhanced the distribution dominance of capital, leading to a higher proportion of corporate profit retention and a lower share of wage income. A decline in the labor income share means that a larger proportion of national income flows to capital factors, and the distribution of capital gains is far more concentrated than that of labor income [59]. The falling labor income share weakens the synchronization between the wage growth of ordinary workers and economic growth, while capital owners reap excess returns through assets such as equities and real estate. This imbalance in the distribution structure of factor incomes is further amplified against the backdrop of aging. Coupled with the labor market segmentation exacerbated by aging itself, it systematically widens the income gap among groups with different ages, skills, and asset levels within cities.

In addition, with the acceleration of the aging process, the proportion of the elderly population in the total population is rising rapidly. After retirement, the elderly group depends to a large extent on the social security system for their economic sources, such as pensions and medical insurance. In order to ensure that the elderly population can maintain a certain standard of living and health status, it is necessary to increase the input of social security accordingly, which leads to an increase in the proportion of social security expenditure in the total fiscal expenditure or the distribution of total social resources [60]. In the social security system, different income groups benefit from social security expenditures to different extents, and there exist certain limitations in the allocation of social security resources. For high-income groups, they typically pay social insurance premiums based on a relatively high wage base during their working period. In terms of pension receipt, high-income individuals receive relatively substantial pension amounts after retirement, which is based on their higher contribution base and longer contribution years. In contrast, low-income groups, due to their lower wage levels and smaller contribution bases, receive relatively meager pension benefits, and the growth rate of their pensions during adjustments is also quite limited. The “fragmented” structure of the social security system, along with the high-income-oriented and urban-biased traits evident in social security expenditures, cause the accessibility and utilization of social security benefits by low-income groups to be markedly weaker than those of middle and high-income groups. Additionally, some high-income individuals may secure even more social security benefits via diverse means [61]. The huge contrast in the degree of benefit from social security expenditures among different income groups enables high-income groups to further consolidate and enhance their wealth accumulation and life security with the assistance of the social security system. In contrast, low-income groups find it difficult to effectively narrow the gap with high-income groups through social security. Instead, with the increase in social security expenditures, the intra-urban income gap shows a further widening tendency.

Based on this, this paper puts forward the following assumptions:

**H3a.** The population aging will expand the intra-urban income gap by reducing the labor income share.

**H3b.** The population aging will expand the intra-urban income gap by increasing social security expenditure.

Population aging will reduce urban technological innovation, thereby widening the inter-urban income gap. Technological innovation activity depends largely on young labor force groups [62]. In regions with a high degree of population aging, the shrinking young population base directly reduces the size of core innovation groups. Meanwhile, the weakened risk-taking capacity of the elderly group and their delayed skill updating lead to a weak local innovation foundation [63]. Meanwhile, the elderly-oriented consumption structure centered on low-elasticity elderly care services inhibits the growth of demand in emerging markets and narrows the space for innovation opportunities. Moreover, local governments, burdened by increasing social security obligations, are forced to reduce investment in technological innovation, further exacerbating the shortage of regional innovation resources and making technological breakthroughs more difficult. In cities with a high degree of population aging and low technological innovation, high-quality jobs continue to be lost amid declining entrepreneurship, restricting employment opportunities for urban residents. This compels young laborers to accelerate their migration to cities with high innovation vitality, forming a vicious cycle of “population outflow—demand contraction—reduced investment—lower income”. In contrast, cities with a lower degree of population aging and high technological innovation, by agglomerating venture capital and high-growth enterprises, continuously strengthen their dominant position in high-value-added industries such as the digital economy and high-end services [64]. As the local economy prospers, residents’ income levels keep rising. Thereby further widening the inter-urban income gap.

At the same time, population aging inhibits regional industrial structure upgrading, systematically widens the generational gaps in industrial structures between cities, and expands the inter-urban income gap. On the one hand, regions with severe aging face a dual pressure of continuous outflow of young technical talents and the delayed skill upgrading of elderly workers, leading to a shrinking supply of high-skilled labor required by advanced manufacturing and modern service industries [1]. Local enterprises, constrained by human capital shortages, struggle to undertake the transfer of technology-intensive industries and lack the capacity to cultivate local innovation clusters, thus being forced into a low-end path lock-in [65]. In contrast, cities with a lower level of population aging, by virtue of the continuous inflow of high-quality labor and advantages in industry-university-research collaboration, accelerate their climb to the upper reaches of the value chain in fields such as the digital economy and high-end equipment manufacturing, forming a positive cycle of human capital agglomeration. On the other hand, as the proportion of the elderly population increases, the demand structure of the consumer market also changes. Elderly people pay more attention to consumption in areas such as healthcare, traditional food, and basic daily necessities, while their demand for emerging consumer electronics, fashion products, and high-end cultural and entertainment products is relatively low. This shift in consumption preferences leads to slow growth in market demand for emerging industries, leaving enterprises with little motivation to carry out product innovation and industrial upgrading [66]. Cities with an advanced industrial structure, relying on sophisticated industrial models, can generate high industrial added value. Their products and services often have substantial profit margins, thus enabling them to offer workers quite considerable incomes. In contrast, cities dominated by traditional industries, constrained by the inherent characteristics of traditional sectors, face meager product profits, resulting in workers’ income levels remaining low for a long time. Such differences in industrial development will further widen the inter-urban income gap.

Based on this, this paper puts forward the following assumptions:

**H4a.** The population aging will expand the inter-urban income gap by reducing technological innovation.

**H4b.** The population aging will expand the inter-urban income gap by hindering industrial structure upgrading.

Digital finance refers to financial activities conducted through digital technologies and internet platforms. It can break through the constraints of physical branches of traditional financial institutions, extending financial services to broader regions and populations. In particular, it provides access to financial services for low-income groups, small and micro enterprises, and residents in remote areas who lack access to traditional financial service channels [67]. With its inclusiveness, efficiency, and precision, digital finance can alleviate the amplifying effect of population aging on intra-urban income gaps through multiple key links. On one hand, digital finance can enhance the labor participation rate of the elderly population. The decline in labor income share caused by population aging is largely attributed to the difficulty for the elderly to participate in market division of labor due to physical conditions and information barriers. However, digital finance, by virtue of mobile payments and big data matching, enables low-skilled elderly people to flexibly engage in part-time work [68]. This “digital gig economy” directly increases the labor income of low-income elderly groups, narrows the gap with high-income groups, and thereby alleviates the impact of population aging on intra-urban income gap. On the other hand, digital finance can narrow the wealth accumulation gap brought about by population aging. Against the backdrop of aging, high-income young groups can achieve wealth appreciation through financial management and investment, while low-income elderly groups, due to a lack of financial literacy and access to small-sum investment channels, are prone to wealth shrinkage. Digital finance lowers the threshold for financial services, enabling low-asset elderly groups—who are excluded by the traditional system—to gain access to channels for asset appreciation [69]. Digital finance not only makes it easier for the elderly to obtain income sources but also ensures, through technical means, that resources are tilted toward vulnerable groups. Ultimately, amid the intensification of aging, it injects momentum for “rebalancing” into intra-urban income distribution.

Based on this, this paper puts forward the following assumptions:

**H5.** Digital finance can alleviate the impact of population aging on the intra-urban income gap.

The development of digital finance can restructure the pattern of regional factor mobility through technological empowerment, activate the economic resilience of underdeveloped regions, and effectively mitigate the effect of population aging on widening inter-urban income gaps. Firstly, digital financial services significantly weaken the geographical dependence of financial resources, enabling regions with a higher degree of aging to break through geographical restrictions. These regions can better obtain cross-regional capital injection by relying on cloud-based financing platforms, optimize cash flow management through digital bill discounting, and reduce credit costs with the help of big data-based risk control, thus partially offsetting the negative impact of local labor force shrinkage and market contraction. Secondly, digital finance can enhance the success rate of entrepreneurship in regions with a higher degree of population aging and create a large number of employment opportunities [70]. It lowers the thresholds for entrepreneurship, enabling entrepreneurs to conveniently access funds, market information, and customer resources through online platforms, which improves the probability of entrepreneurial success [71]. This drives employment and economic development, increases residents’ income, providing strong support for the economic recovery and sustainable development of aging regions. Finally, digital finance helps unleash the potential of the “silver economy” in aging cities. By building a “payment-wealth management-consumption” closed loop, digital finance activates the consumption and investment demands of the elderly group [72]. The rise of the silver economy not only creates new economic growth points but also forms a positive cycle of “aging forcing industrial innovation”, injecting sustainable momentum into urban economies. This thus weakens the rigid constraints of population aging on urban economic development and income levels.

Based on this, this paper puts forward the following assumptions:

**H6.** Digital finance can alleviate the impact of population aging on the inter-urban income gap.

**Fig 1:**
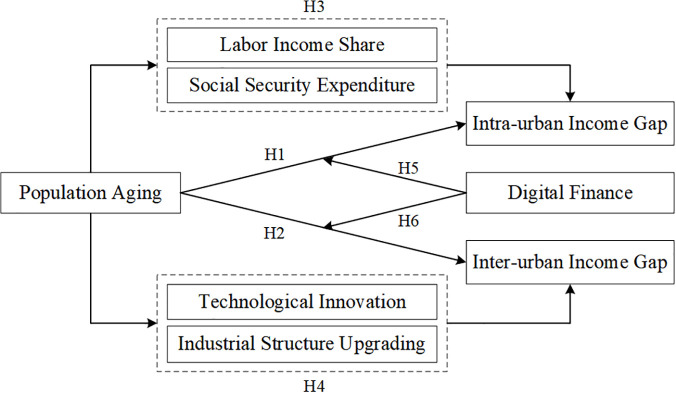
Research framework.

The proposed model of this article is shown in Fig 1.

## Research methodology

### Model specification

In order to test the impact of population aging on income gap, the following econometric models are constructed based on the above theoretical analysis:


$$InGap_{\;i,\;t}=\beta_{\text{0}}+\beta_{\text{1}}Agi_{\;i,\;t}+\sum_{k}\beta_{k}Controls_{\;i,\;t}+\mu_{i}+\delta_{t}+\varepsilon_{i,\;t}$$ (1)

$$ExGap_{\;i,\;t}=\beta_{\text{0}}+\beta_{\text{1}}Agi_{\;i,\;t}+\sum_{k}\beta_{k}Controls_{\;i,\;t}+\mu_{i}+\delta_{t}+\varepsilon_{i,\;t}$$ (2)

In Equation (1) and Equation (2), InGap_i,t_ denotes the intra-urban income gap of city i in year t, ExGap_i,t_ denotes the inter-urban income gap of city i in year t, and Agi_i,t_ denotes the population aging of city i in year t. Controls are a set of control variables. β_1_ is the regression coefficient of the effect of population aging on income gap, β_k_ (k ≠ 0,1) is the regression coefficient of control variables, μ_i_ and δ_t_ represent Fixed region effects and Fixed time effects, respectively, and ε_i,t_ denotes the random error term.

Building upon the aforementioned theoretical analysis, in order to gain a more in-depth understanding of the mechanism by which population aging affects the income gap, the following econometric model is developed for empirical examination.


$$Med1_{\;i,\;t}=\alpha_{\text{0}}+\alpha_{\text{1}}Agi_{\;i,\;t}+\sum_{k}\alpha_{k}Controls_{\;i,\;t}+\mu_{i}+\delta_{t}+\varepsilon_{\;i,\;t}$$ (3)

$$InGap_{\;i,\;t}=\alpha_{\text{0}}+\alpha_{\text{1}}Med1_{\;i,\;t}+\alpha_{\text{2}}Agi_{\;i,\;t}+\sum_{k}\alpha_{k}Controls_{\;i,\;t}+\mu_{i}+\delta_{t}+\varepsilon_{\;i,\;t}$$ (4)

$$Med2_{\;i,\;t}=\alpha_{\text{0}}+\alpha_{\text{1}}Agi_{\;i,\;t}+\sum_{k}\alpha_{k}Controls_{\;i,\;t}+\mu_{i}+\delta_{t}+\varepsilon_{\;i,\;t}$$ (5)

$$ExGap_{\;i,\;t}=\alpha_{\text{0}}+\alpha_{\text{1}}Med2_{\;i,\;t}+\alpha \text{2}Agi_{\;i,\;t}+\sum_{k}\alpha_{k}Controls_{\;i,\;t}+\mu_{i}+\delta_{t}+\varepsilon_{\;i,\;t}$$ (6)

Med1_i,t_ represents the mechanism variables of population aging affecting the intra-urban income gap, including labor income share(LIS) and social security expenditure(SSE). Med2_i,t_ denotes the mechanism variables that population aging affects the inter-urban income gap, including technological innovation(TI) and industrial structure upgrading(ISU). α is the regression coefficient.

In order to solve the problem of income gap caused by population aging that can be alleviated by digital finance, this article introduces the interaction term between digital finance and population aging, and constructs a regression model containing the moderating effect as shown below.


$$InGap_{\;i,\;t}=\gamma_{\text{0}}+\gamma_{\text{1}}Agi_{\;i,\;t}+\gamma_{\text{2}}Index_{\;i,\;t}+\gamma_{\text{3}}Index_{\;i,\;t}\times Agi_{\;i,\;t}+\sum_{k}\gamma_{k}Controls_{\;i,\;t}+\mu_{i}+\delta_{t}+\varepsilon_{\;i,\;t}$$ (7)

$$ExGap_{\;i,\;t}=\gamma_{\text{0}}+\gamma_{\text{1}}Agi_{\;i,\;t}+\gamma_{\text{2}}Index_{\;i,\;t}+\gamma_{\text{3}}Index_{\;i,\;t}\times Agi_{i,t}+\sum_{k}\gamma_{k}Controls_{\;i,\;t}+\mu_{i}+\delta_{t}+\varepsilon_{\;i,\;t}$$ (8)

In Equation (7) and Equation (8), Index_i,t_ denotes the level of digital finance(DFI) in city i in year t. γ is the regression coefficient.

### Measures of variables

#### Independent variables.

**Intra-urban Income Gap(InGap)**. Existing studies generally use the Gini coefficient to express the intra-urban income gap. The Gini coefficient is a commonly used indicator for measuring the fairness of income distribution among residents, ranging between 0 and 1. The higher the Gini coefficient, the greater the intra-urban income gap.

**Inter-urban Income Gap(ExGap)**. Measured by the ratio of the maximum annual national average income to the annual average income of the city. The larger the ratio, the greater the income gap between the city and the most developed city. The maximum annual average income in the country represents the “ceiling” of the domestic income level during the same period, and calculating the ratio based on it can eliminate the problem of different reference standards in different regions and at different times.

#### Dependent variables.

**Population Aging (Agi)**. The international common standard defines the level of population aging as the proportion of population over 65 years old. Referring to the research of Zhang et al. [73], Feng & Li [74], we used the database of the sixth national population census in 2010 and the seventh national population census in 2020, and the national 1% population sampling survey in 2005 and 2015, and used the interpolation method to calculate the regional population aging level. The change in population age structure is gradual, and there will not be drastic fluctuations in the short term. Therefore, linear interpolation can reasonably approximate the true trend.

#### Mediator variables.

**Labor Income Share(LIS)**. The labor income share reflects the income distribution proportion of workers in the value created by regional economic production activities, and is measured by the ratio of the total annual wage compensation to the total GDP in the region.

**Social Security Expenditure(SSE)**. The social security expenditure is expressed as the ratio of local government’s total social security expenditure to total GDP.

**Technological Innovation(TI)**. Patents represent the institutional output of innovative activities, and the volume of patent output directly reflects the knowledge creation intensity in a region. To avoid measurement bias caused by examination lags and more timely capture innovation dynamics, this paper uses the natural logarithm of the annual number of patent applications in each region to measure the level of regional technological innovation.

**Industrial Structure Upgrading(ISU)**. Based on the realistic background of macro- economic growth and industrial structure adjustment, Clark theorem believes that the manifestation of industrial structure development is the gradual upgrading of regional leading industries, that is, the process of structural focus transfer from the primary industry to the secondary industry and then gradually to the tertiary industry. Therefore, combined with the reality of China, this paper constructs a comprehensive industrial structure upgrading index to measure the level of regional industrial structure upgrading.


$$Indus\;i,t=\sum_{k=\text{1}}^{\text{3}}Yi,k,t\times k$$ (9)

Indus_i,t_ indicates the industrial structure upgrading of city i in year t. k = 1,2,3 refer to primary industry, secondary industry and tertiary industry, respectively. Y_i,k,t_ indicates the proportion of the output value of the k-th industry of city i in the regional GDP in year t. The larger the index, the higher the level of industrial structure upgrading.

#### Moderator variable.

**Digital Finance**. Digital finance is gauged by the Digital Financial Inclusion Index developed by the Digital Finance Research Centre of Peking University. This index encompasses the overall Digital Financial Inclusion Index (DFI) as well as multiple sub-indices. Specifically, these sub-indices include the Breadth of Digital Financial Inclusion Coverage (Bread), the Depth of Digital Financial Inclusion Usage (Depth), and the Degree of Digitisation of Digital Financial Inclusion (Digit).

#### Control variables.

In view of other factors that may have an impact on the urban income gap, this paper selects the following indicators as control variables to be included in the regression model. This paper selects foreign investment level, financial development level, healthcare infrastructure, and industrial automation development level as control variables to explore the impact of population aging on the intra-urban and inter-urban income gaps. (1)Foreign investment (Invest). The level of foreign investment affects employment and income distribution, and its distribution differences may exacerbate the income gap between cities. This paper uses the ratio of the number of foreign-invested enterprises in cities to industrial enterprises above designated size to measure the level of foreign investment. (2)Financial development (Finan). The level of financial development will affect income gap through resource allocation. This paper uses the ratio of the total amount of loans at the end of the period to the total GDP of the region to represent it. (3)Healthcare infrastructure (Health). Healthcare infrastructure is directly related to aging, and its level of improvement affects income structure and the development gap. Refer to the study by Garcia-Mila & McGuire [75], this paper uses the number of medical beds per 10000 people to measure the level of regional healthcare infrastructure. (4) Industrial automation levels(Automa). The level of industrial automation is both driven by aging population and directly affects income distribution. Referring to the research of Acemoglu & Stresepo [56], we use the number of imported robots to measure the level of industrial automation development in a region.

### Data description

In an effort to strike a balance between data availability and completeness, this article designates 284 prefecture-level cities in China as the research subjects, with the sample period ranging from 2011 to 2022. The data primarily originate from the statistical yearbooks of each prefecture-level city spanning from 2011 to 2022, publicly available data on the Internet, local government websites, and other information query platforms. The detailed raw dataset is provided in S1 File. The indicators involving prices are converted with 2011 as the base period. The descriptive statistical results are shown in Table 1, which reports the number of observations, mean, standard deviation, minimum and maximum values of the main variables, and can intuitively reflect the overall distribution characteristics of the data. The data verification results show that there are no extreme outliers in all the data.

**Table 1:** Descriptive statistics.

Variables	N	Mean	Std.deviation	Min	Max
InGap	3408	0.524	0.158	0.233	0.849
ExGap	3408	2.226	0.421	1.000	4.404
Agi	3408	0.123	0.032	0.019	0.237
LIS	3408	0.125	0.045	0.036	0.496
SSE	3408	0.028	0.020	0.002	0.208
TI	3408	8.180	1.684	0.690	12.470
ISU	3408	2.308	0.143	1.831	2.836
DFI	3408	5.155	0.524	2.830	5.890
Bread	3408	5.104	0.591	0.620	5.970
Depth	3408	5.117	0.510	1.460	5.870
Digit	3408	5.301	0.591	0.993	6.365
Invest	3408	0.040	0.048	0.000	0.335
Finan	3408	1.068	0.629	0.118	5.797
Health	3408	0.477	0.178	0.135	1.377
Automa	3408	0.153	0.354	0.001	5.135

To ensure the applicability of panel data for subsequent econometric analysis, this paper uses the LM test and LLC test to verify variable stationarity. As shown in Table 2, both tests yield p-values below 0.01, significantly rejecting the null hypothesis of “presence of unit roots”, indicating that all variables pass the stationarity test with no unit root issues. This result provides a data foundation for subsequent model estimation, avoiding spurious regression caused by non-stationary data and ensuring the reliability of empirical results.

**Table 2:** Time series stationarity test of main variables.

Variables	N	LM Test		LLC Test	
		z_Statistic	p-value	Adj_t	p-value
InGap	3408	12.865	0.000	−10.789	0.000
ExGap	3408	29.692	0.000	−22.069	0.000
Agi	3408	15.993	0.000	−172.296	0.000
LIS	3408	24.320	0.000	−35.744	0.000
SSE	3408	31.836	0.000	−13.406	0.000
TI	3408	17.914	0.000	−9.322	0.000
ISU	3408	6.579	0.000	−6.846	0.000
DFI	3408	51.082	0.000	−19.223	0.000
Bread	3408	42.805	0.000	−6.706	0.000
Depth	3408	45.552	0.000	−3.229	0.000
Digit	3408	43.972	0.000	−61.254	0.000
Invest	3408	9.553	0.000	−17.382	0.000
Finan	3408	42.177	0.000	−10.788	0.000
Health	3408	19.414	0.000	−22.291	0.000
Automa	3408	56.300	0.000	−31.356	0.000

Table 3 reports the correlation analysis results of the main variables, which show that the dependent variable InGap is significantly negatively correlated with the independent variable Agi, preliminarily supporting the research hypothesis. However, the dependent variable ExGap is significantly negatively correlated with the independent variable Agi, contrary to the expected direction of the research hypothesis. The correlation between other variables is basically as expected, and no severe multicollinearity was found between variables (VIF test results showed that the VIF values of each variable were far below 10). To obtain more robust data analysis results, further validation through subsequent regression analysis is necessary.

**Table 3:** Correlation analysis of main variables.

	1	2	3	4	5	6	7	8	9	10	11	12	13	14	15
InGap	1.000														
ExGap	0.165***	1													
Agi	−0.166***	0.272***	1.000												
LIS	−0.205***	−0.236***	−0.118***	1.000											
SSE	0.148***	0.392***	0.270***	0.271***	1.000										
TI	−0.349***	−0.489***	−0.052***	0.121***	−0.497***	1.000									
ISU	−0.480***	−0.458***	0.046***	0.359***	−0.233***	0.551***	1.000								
DFI	−0.525***	−0.049***	0.461***	0.257***	0.032*	0.249***	0.499***	1.000							
Bread	−0.519***	−0.094***	0.426***	0.262***	−0.016	0.272***	0.532***	0.976***	1.000						
Depth	−0.536***	−0.029*	0.482***	0.213***	0.022	0.279***	0.465***	0.960***	0.905***	1.000					
Digit	−0.375***	0.071***	0.421***	0.223***	0.147***	0.076***	0.318***	0.899***	0.822***	0.846***	1.000				
Invest	−0.243***	−0.429***	−0.117***	0.197***	−0.229***	0.466***	0.408***	0.032*	0.078***	0.043**	−0.118***	1.000			
Finan	−0.290***	−0.357***	−0.020	0.576***	0.038**	0.325***	0.565***	0.373***	0.399***	0.325***	0.247***	0.262***	1.000		
Health	−0.219***	−0.338***	−0.023	0.395***	−0.131***	0.313***	0.518***	0.365***	0.413***	0.292***	0.238***	0.349***	0.562***	1.000	
Automa	−0.300***	−0.299***	0.044**	0.286***	−0.146***	0.502***	0.422***	0.330***	0.328***	0.332***	0.235***	0.354***	0.303***	0.378***	1.000
VIF	–	1.72	1.69	2.07	1.71	1.75	2.35	2.18	–	–	–	1.47	2.29	1.77	1.58

## Empirical results and discussion

### Baseline modelling results

Table 4 uses a fixed effects model to estimate the impact of population aging on intra-urban income gap and inter-urban income gap. The Columns (1) and (2) respectively explore the impact of population aging on intra-urban income gap and inter-urban income gap without controlling for variables. It is shown that the impact of population aging on both the intra-urban income gap and inter-urban income gap is significantly positive at the 1% statistical level, suggesting that population aging will increase the intra-urban income gap and inter-urban income gap. On this basis, control variables are incorporated in Columns (3) and (4), and the findings remain significantly positive. The result provides strong evidence for H1 and H2.

**Table 4:** Benchmark regression results.

	(1)	(2)	(3)	(4)
	InGap	ExGap	InGap	ExGap
Agi	1.359*** (0.095)	5.363*** (0.280)	1.240*** (0.104)	2.194*** (0.868)
Invest			−0.648*** (0.052)	−1.273*** (0.142)
Finan			0.003 (0.004)	−0.066*** (0.011)
Health			−0.037*** (0.014)	−0.466*** (0.040)
Automa			0.040*** (0.007)	−0.104*** (0.018)
Cons	0.411*** (0.030)	0.496*** (0.088)	0.525*** (0.032)	1.545*** (0.089)
Fixed region	Yes	Yes	Yes	Yes
Fixed time	Yes	Yes	Yes	Yes
N	3408	3408	3408	3408
R^2^	0.620	0.541	0.644	0.621

Note: ***, **, and * represent significance at the levels of 1%, 5%, and 10%, respectively. The values in parentheses are standard errors. The same below.

### Robustness analyses

#### Replace the dependent variable.

To guarantee the robustness of the research results and circumvent biases in the outcomes stemming from improper variable measurement methods, the Theil Index is adopted as a substitute for the intra-urban income gap. Simultaneously, the ratio of the maximum annual urban per capita GDP to the urban per capita GDP is utilized as a proxy for the inter-urban income gap to carry out the robustness test. The specific regression results are shown in Column (1) of Table 5. It can be observed that after replacing the dependent variables, the impact of population aging on both intra-urban income gap and inter-urban income gap remains significantly positive at the 1% statistical level. Therefore, the above benchmark regression results are robust.

**Table 5:** Robustness test results.

	(1)		(2)		(3)		(4)	
	Replace dependent variable		Replace independent variable		Exclude provincial capital cities		GMM model	
	InGap	ExGap	InGap	ExGap	InGap	ExGap	InGap	ExGap
Agi	0.052*** (0.005)	5.523*** (1.847)	0.643*** (0.126)	1.748*** (0.344)	1.265*** (0.110)	1.753*** (0.296)	1.282*** (0.350)	2.792** (1.059)
Invest	−0.015*** (0.002)	−6.640*** (0.913)	−0.643*** (0.053)	−1.268*** (0.142)	−0.630*** (0.056)	−1.164*** (0.150)	−1.345*** (0.132)	−2.634*** (0.416)
Finan	0.001*** (0.0002)	0.766*** (0.073)	0.001 (0.004)	−0.072*** (0.011)	0.013* (0.007)	0.012 (0.018)	0.010 (0.010)	−0.066 (0.053)
Health	−0.002*** (0.0007)	−7.596*** (0.255)	−0.082*** (0.014)	−0.543*** (0.038)	−0.031* (0.016)	−0.436*** (0.043)	0.050 (0.042)	−0.196 (0.161)
Automa	0.007*** (0.0003)	0.537*** (0.117)	0.023*** (0.007)	−0.134*** (0.018)	0.045*** (0.009)	−0.073*** (0.024)	0.018 (0.012)	−0.201*** (0.036)
Cons	0.046*** (0.002)	6.199*** (0.572)	0.617*** (0.033)	1.644*** (0.089)	0.468*** (0.017)	2.265*** (0.046)	0.540*** (0.041)	2.189*** (0.157)
Fixed region	Yes	Yes	Yes	Yes	Yes	Yes	Yes	Yes
Fixed time	Yes	Yes	Yes	Yes	Yes	Yes	Yes	Yes
N	3408	3408	3408	3408	3036	3036	3408	3408
R^2^	0.980	0.524	0.632	0.617	0.640	0.571	–	–
F	–	–	–	–	–	–	70.29	40.65
AR(1)	–	–	–	–	–	–	0.000	0.000
AR(2)	–	–	–	–	–	–	0.523	0.526
Hansen	–	–	–	–	–	–	0.879	0.867

#### Replace the independent variable.

To avoid the deficiency of using only the proportion of elderly population to reflect the scale and neglecting the support capacity of the working age population, the dependency ratio of elderly population aged 65 and above in various regions is used as a substitute indicator for population aging for robustness testing. The results are presented in Column (2) of Table 5. It can be seen that after replacing the independent variables, the impact of population aging on intra-urban income gap and inter-urban income gap is still significantly positive, so the above findings pass the robustness test.

#### Exclude provincial capital cities.

To prevent estimation bias triggered by the resource imbalance between provincial capital cities and other prefecture-level cities, this paper excludes 31 provincial capital cities (or municipalities directly under the central government) from the 284 sample cities for the regression analyses, and the specific regression results are shown in Column (3) of Table 5. It can be clearly seen that after excluding the data of provincial capital cities, population aging still significantly and positively affects the intra-urban and inter-urban income gaps, indicating that the regression results remain robust.

#### GMM model.

To avoid interference from endogeneity and model specification biases on the reliability of conclusions, this paper employs the GMM model for robustness checks, with results presented in Column 4 of Table 5. The GMM robustness test results indicate that the impact of population aging on both intra-urban and inter-urban income gaps is significantly positive at a statistical significance level of at least 5%. Meanwhile, the AR(1) test yields a p-value of 0.000, and the AR(2) test yields a p-value of 0.526, confirming the validity of the model’s dynamic specification. The Hansen test yields a p-value of 0.867, failing to reject the null hypothesis, which indicates that the selection of instrumental variables is effective and satisfies the exogeneity requirement. These test results collectively validate the effectiveness of the GMM estimation, further confirming the robustness of the core conclusions.

#### Instrumental variable method.

Regarding the endogeneity issue that the regression results might encounter, this paper adopts the instrumental variable method to test the endogeneity of the baseline regression. Drawing on the relevant research of Acemoglu & Restrepo [56], this study uses the crude birth rate of various regions 65 years ago (1946–1957) as an instrumental variable for population aging. The rationality of selecting instrumental variables lies in: Firstly, the crude birth rate in various regions from 1946 to 1957 directly determines the size of the elderly population 65 years later (2011–2022), which conforms to the queue effect of population age evolution. Secondly, the birth rate 65 years ago was mainly determined by the socio-economic conditions at that time, which were not directly related to current economic and social factors and met exclusivity constraints. Finally, the birth rate 65 years ago as a historical variable cannot be negatively affected by the current level of aging, thus avoiding the problem of reverse causality. Based on this, this papaer uses two-stage least squares method for regression analysis, and the results are shown in Table 6. It can be observed that the results obtained from the instrumental variable method analysis indicate that the impact of population aging on both the intra-urban income gap and inter-urban income gap is significantly positive. Thus, the original conclusion withstands the robustness test.

**Table 6:** Results of instrumental variable analysis.

	(1)	(2)	(3)
	Agi	InGap	ExGap
	First stage	Second stage	Second stage
IV_Agi	0.014*** (0.003)		
Agi		18.530*** (5.239)	6.293** (2.881)
Invest	0.020 (0.040)	−0.805 (0.629)	−1.310*** (0.382)
Finan	−0.002 (0.002)	0.031 (0.037)	−0.059 (0.042)
Health	−0.035*** (0.011)	0.597** (0.289)	−0.316 (0.217)
Automa	−0.014*** (0.004)	0.281*** (0.092)	−0.046 (0.044)
Cons	0.098*** (0.012)	−1.681** (0.702)	1.022*** (0.348)
Fixed region	Yes	Yes	Yes
Fixed time	Yes	Yes	Yes
N	3408	3408	3408
R^2^	0.772	0.649	0.603

### Heterogeneity analyses

#### Heterogeneity of urban hierarchical.

To delve into the disparities in the influence of population aging on the urban income gap across different city tiers, this study draws on the classification of city levels in the “2020 China City Business Charm Ranking”. First-tier, second-tier, and third-tier cities are categorized as developed cities(DC), while fourth-tier and fifth-tier cities are grouped as underdeveloped cities(UDC). Group regressions are then conducted, and the results are presented in Columns (1) and (2) of Table 7. It is evident that within both the sample of developed cities and underdeveloped cities, population aging has a significant positive impact on the intra-urban income gap. It can be seen from the inter-group coefficient that there is no significant difference between the two. For developed cities, the impact of population aging on the inter-urban income gap is not significant, while for underdeveloped cities, population aging will significantly increase the inter-urban income gap. The underlying cause is that the economic structures of developed cities are more diversified and resilient. New industries and service sectors are booming, and these cities typically have well-developed financial, scientific and technological, and high-end manufacturing industries. Their degree of dependence on the quantity of laborers is relatively low. Therefore, when confronted with population aging, the economic system can be adjusted relatively flexibly. This flexibility allows them to mitigate the impact of population aging on the inter-urban income gap. In the underdeveloped regions, the industrial structure is mainly characterized by traditional industries, which are relatively homogeneous. The labor shortage resulting from population aging will have a more substantial impact on these industries. Low-income groups that rely on manual labor and traditional industries may encounter greater employment pressures, leading to slow or even stagnant income growth. In contrast, high-skilled and high-income groups will be relatively less affected. As a consequence, the inter-urban income gap in these regions is further widened.

**Table 7:** Regression results of heterogeneity analysis.

	(1)		(2)		(3)		(4)	
	InGap		ExGap		InGap		ExGap	
	DC	UDC	DC	UDC	RBC	NRBC	RBC	NRBC
Agi	0.814*** (0.140)	0.799*** (0.161)	0.329 (0.366)	3.116*** (0.437)	0.912*** (0.202)	1.134*** (0.137)	4.285*** (0.648)	0.752** (0.332)
Invest	−0.414*** (0.054)	−0.396*** (0.126)	−1.077*** (0.140)	−0.084 (0.342)	0.051 (0.175)	−0.590*** (0.062)	0.137 (0.561)	−1.307*** (0.150)
Finan	−0.014*** (0.005)	0.026*** (0.008)	−0.225*** (0.014)	0.105*** (0.023)	0.053*** (0.009)	−0.009* (0.005)	0.132*** (0.031)	−0.124*** (0.013)
Health	−0.092*** (0.019)	0.001 (0.021)	−0.344*** (0.049)	−0.565*** (0.059)	−0.020 (0.025)	−0.025 (0.020)	−0.518*** (0.082)	−0.501*** (0.048)
Automa	0.028*** (0.006)	0.111** (0.054)	−0.105*** (0.016)	−0.609*** (0.148)	0.176*** (0.045)	0.033*** (0.007)	−0.143 (0.142)	−0.095*** (0.017)
Cons	0.567*** (0.031)	0.553*** (0.025)	1.870*** (0.082)	2.078*** (0.067)	0.478*** (0.025)	0.533*** (0.036)	1.912*** (0.079)	1.826*** (0.086)
Fixed region	Yes	Yes	Yes	Yes	Yes	Yes	Yes	Yes
Fixed time	Yes	Yes	Yes	Yes	Yes	Yes	Yes	Yes
N	1392	2016	1392	2016	1380	2028	1380	2028
R^2^	0.679	0.655	0.779	0.535	0.701	0.630	0.460	0.725
Diff. p-value	0.358		0.000		0.168		0.000	

#### Heterogeneity of resource endowments.

To explore the differences in the impact of population aging on urban income gaps under diverse resource endowments, in accordance with the definition of resource cities in the “National Sustainable Development Plan for Resource Cities (2013-2020)” issued by the State Council of China, the sample cities are classified into resource-based cities(RBC) and non-resource-based cities(NRBC), and group regressions are carried out. The results are presented in Columns (3) and (4) of Table 7. It can be observed that population aging has a significant positive impact on the intra-urban and inter-urban income gaps in both resource-based and non-resource-based cities. The impact of population aging on inter-urban income gap is more significant in resource-based cities. The reason behind this is that resource-based cities are highly economically dependent on the extraction of mineral, energy and other resources, with a single industrial structure that has a high demand for manual labor. Population aging has led to a reduction in the supply of young and middle-aged labor, directly impacting the production capacity of resource industries and causing a slowdown in income growth. At the same time, the transformation of resource-based cities is generally lagging behind, with insufficient development of emerging industries. Aging further weakens innovation vitality and transformation momentum, making it difficult to make up for the income gap in resource industries through diversified industries. Non resource-based cities can absorb surplus labor through diversified industries such as service and manufacturing, and cushion the income impact of aging.

### Mechanism analyses

Table 8 reports the regression results of the impact mechanism of population aging on the intra-urban income gap. As can be observed from Columns (1) and (2) of Table 8, population aging has a significantly negative impact on the labor income share at the 10% statistical significance level. Moreover, the labor income share exerts a negative effect on the intra-urban income gap. This indicates that population aging widens the intra-urban income gap by reducing the urban labor income share. Table 9 presents the results of the mediation effect test using Bootstrap method, with a randomly selected sample size of 1000. When using the Bootstrap method to determine whether the mediation effect is significant, it is necessary to observe whether the interval between LLCI and ULCI contains 0. If 0 is not included, it means that the mediation effect is significant, otherwise it is not significant. The results show that the mediating effect of population aging on intra-urban income gap through labor income share is within the range of [0.0003, 0.010], which clearly does not include 0. This confirms that population aging can indeed affect intra-urban income gap by influencing labor income share. Therefore, it is assumed that H3a passes the verification. Simultaneously, Columns (3) and (4) of Table 8 demonstrate that population aging significantly boosts urban social security expenditures. The effect of social security expenditures on intra-urban income gap is significantly positive. The Bootstrap test results show that the intermediate transmission range of the impact of population aging on intra-urban income gap through social security expenditure also does not include 0, indicating that the mediating effect of social security expenditure is significant. Therefore, H3b is verified.

**Table 8:** Mechanism regression results of population aging on intra-urban income gap.

	(1)	(2)	(3)	(4)
	LIS	InGap	SSE	InGap
Agi	−0.054* (0.032)	1.234*** (0.204)	0.184*** (0.015)	1.194*** (0.107)
LIS		−0.099* (0.055)		
SSE				0.240** (0.117)
Invest	0.049*** (0.016)	−0.643*** (0.052)	−0.012 (0.007)	−0.645*** (0.051)
Finan	0.030*** (0.001)	0.006 (0.004)	0.005*** (0.001)	0.002 (0.004)
Health	−0.007 (0.004)	−0.038*** (0.014)	−0.036*** (0.002)	−0.029* (0.015)
Automa	0.013*** (0.002)	0.042*** (0.007)	0.001 (0.001)	0.040*** (0.007)
Cons	0.258*** (0.010)	0.551*** (0.035)	0.021*** (0.005)	0.520*** (0.032)
Fixed region	Yes	Yes	Yes	Yes
Fixed time	Yes	Yes	Yes	Yes
N	3408	3408	3408	3408
R^2^	0.577	0.645	0.524	0.645

**Table 9:** Mediation analysis of population aging on intra-urban income gap by Bootstrap.

Mediator variable	Effect	Coef	Std_err	z	P > |z|	LLCI	ULCI
LIS	Indirect Effect	0.005	0.025	2.08	0.037	0.0003	0.010
	Direct Effect	1.234	0.128	9.62	0.000	0.982	1.485
	Total Effect	1.239	0.127	9.76	0.000	0.990	1.488
SSE	Indirect Effect	0.044	0.013	3.31	0.001	0.018	0.071
	Direct Effect	1.195	0.082	14.61	0.000	1.035	1.355
	Total Effect	1.239	0.077	16.14	0.000	1.089	1.390

Table 10 reports the regression results of the impact mechanism of population aging on the urban inter-urban income gap. As indicated by Columns (1) and (2), population aging exerts a significant negative influence on technological innovation. Additionally, technological innovation significantly mitigates the urban inter-urban income gap. This implies that population aging diminishes urban technological innovation, thereby increasing the inter-urban income gap. Columns (3) and (4) demonstrate that, at the 1% statistical significance level, population aging has a significant negative effect on the industrial structure upgrading. Moreover, the industrial structure upgrading significantly reduces the inter-urban income gap. This indicates that population aging can widen the inter-urban income gap in cities by impeding the industrial structure upgrading. Meanwhile, further verification of the mediating effect using Bootstrap method (as shown in Table 11) reveals that the mediating effect of technological innovation and industrial structure upgrading is significant. Therefore, H4a and H4b are verified.

**Table 10:** Mechanism regression results of population aging on inter-urban income gap.

	(1)	(2)	(3)	(4)
	TI	ExGap	ISU	ExGap
Agi	−4.563*** (1.011)	1.969*** (0.283)	−0.178* (0.009)	2.068*** (0.280)
TI		−0.049*** (0.005)		
ISU				−0.703*** (0.053)
Invest	5.771*** (0.500)	−0.988*** (0.142)	0.478*** (0.045)	−0.936*** (0.140)
Finan	0.471*** (0.040)	−0.043*** (0.011)	0.058*** (0.004)	−0.025** (0.011)
Health	1.944*** (0.140)	−0.370*** (0.040)	0.194*** (0.013)	−0.329*** (0.040)
Automa	0.613*** (0.064)	−0.074*** (0.018)	0.001 (0.006)	−0.104*** (0.018)
Cons	8.446*** (0.313)	1.961*** (0.096)	2.189*** (0.030)	3.420*** (0.147)
Fixed region	Yes	Yes	Yes	Yes
Fixed time	Yes	Yes	Yes	Yes
N	3408	3408	3408	3408
R^2^	0.706	0.637	0.666	0.640

**Table 11:** Mediation analysis of population aging on inter-urban income gap by Bootstrap.

Mediator variable	Effect	Est	Std_err	z	P > |z|	LLCI	ULCI
EA	Indirect Effect	0.225	0.014	15.82	0.000	0.197	0.253
	Direct Effect	1.969	0.157	12.56	0.000	1.662	2.276
	Total Effect	2.194	0.273	14.50	0.000	1.897	2.491
ISU	Indirect Effect	0.125	0.035	3.60	0.000	0.057	0.194
	Direct Effect	2.068	0.110	18.89	0.000	1.854	2.283
	Total Effect	2.194	0.076	28.72	0.000	2.044	2.344

### Regulatory effect analyses

Table 12 shows the results concerning the moderating function of digital financial inclusion and its sub-dimensions within the relationship between population aging and the income gaps. From Columns (1) and (2), it can be seen that the coefficients of the interaction term between population aging and digital inclusive finance on both intra-urban and inter-urban income gaps are significantly negative at the 1% level, indicating that digital inclusive finance can significantly negatively regulate the impact of population aging on the intra-urban and inter-urban income gaps. Columns (3) to (8) report on the moderating effect of the sub items of digital inclusive finance. It can be seen that the breadth, depth, and degree of digital transformation of digital inclusive finance play a significant negative moderating role between population aging and the intra-urban and inter-urban income gaps. Therefore, H5 and H6 have been validated.

**Table 12:** Regression results of regulatory effect.

	(1)	(2)	(3)	(4)	(5)	(6)	(7)	(8)
	InGap	ExGap	InGap	ExGap	InGap	ExGap	InGap	ExGap
Agi	5.058*** (0.845)	10.911*** (2.238)	4.227*** (0.777)	11.791*** (2.058)	6.533*** (0.880)	14.770** (2.417)	4.694*** (0.747)	11.286*** (2.238)
DFI	−0.006 (0.023)	−0.667*** (0.062)						
Bread			0.028* (0.016)	−0.226*** (0.042)				
Depth					0.022 (0.018)	0.026 (0.050)		
Digit							0.073*** (0.016)	0.089* (0.048)
Agi × DFI	−0.705*** (0.154)	−1.626*** (0.409)						
Agi×Bread			−0.556*** (0.142)	−1.823*** (0.378)				
Agi×Depth					−0.975*** (0.161)	−2.314*** (0.444)		
Agi×Digit							−0.624*** (0.134)	−1.072*** (0.402)
Invest	−0.574*** (0.053)	−0.601*** (0.142)	−0.606*** (0.053)	−0.773*** (0.140)	−0.571*** (0.053)	−1.071*** (0.145)	−0.629*** (0.052)	−1.264*** (0.142)
Finan	0.005 (0.004)	−0.039*** (0.011)	0.004 (0.004)	−0.050*** (0.011)	0.005 (0.004)	−0.062*** (0.011)	0.003 (0.004)	−0.066*** (0.011)
Health	−0.012 (0.015)	−0.224*** (0.041)	−0.021 (0.015)	−0.257*** (0.041)	−0.019 (0.015)	−0.419*** (0.040)	−0.033** (0.014)	−0.464*** (0.040)
Automa	0.036*** (0.007)	−0.145*** (0.018)	0.037*** (0.007)	−0.153*** (0.018)	0.037*** (0.007)	−0.112*** (0.018)	0.039*** (0.007)	−0.104*** (0.018)
Cons	0.438*** (0.110)	3.837*** (0.291)	0.331*** (0.085)	2.068*** (0.224)	0.301*** (0.093)	1.115*** (0.256)	0.143 (0.088)	1.372*** (0.242)
Fixed region	Yes	Yes	Yes	Yes	Yes	Yes	Yes	Yes
Fixed time	Yes	Yes	Yes	Yes	Yes	Yes	Yes	Yes
N	3408	3408	3408	3408	3408	3408	3408	3408
R^2^	0.648	0.651	0.646	0.654	0.654	0.625	0.647	0.621

**Fig 2:**
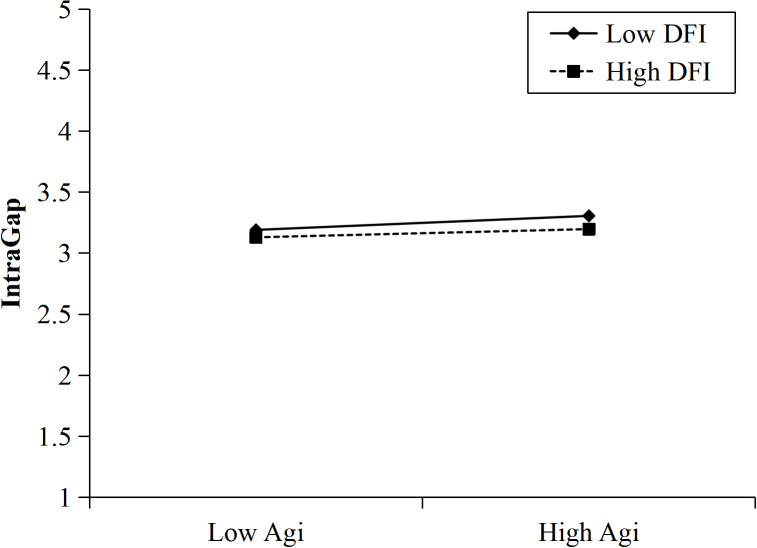
The moderating effect of digital finance differences on population aging and intra-urban income gap.

Fig 2 shows the moderating effect of digital finance on population aging and the intra-urban income gap. It can be seen that in the research scenario of intra-urban income gap (InGap), the development level of digital finance (DFI) exhibits a significant moderating effect. When DFI is at a low level, the positive expansion effect of Agi on InGap is relatively prominent. In the high DFI group, the increase in InGap significantly narrowed during the aging process. This intuitively confirms that digital finance can significantly alleviate the positive impact of population aging on the intra-urban income gap.

**Fig 3:**
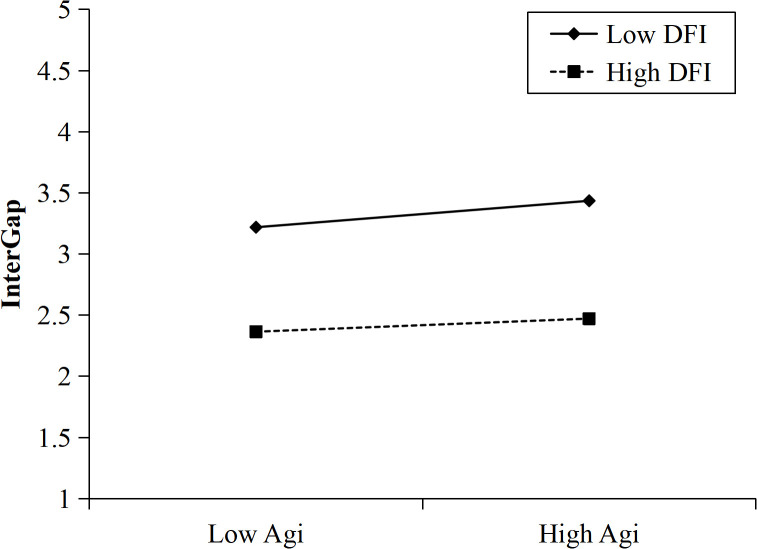
The moderating effect of digital finance differences on population aging and inter-urban income gap.

Fig 3 shows the moderating effect of digital finance on the relationship between population aging and the inter-urban income gap. It can be seen that digital finance(DFI) has a negative moderating effect on the relationship between population aging(Agi) and the inter-urban income gap(ExGap). Under low DFI, the increase in Agi has significantly expanded the ExGap. In high DFI, the positive impact of Agi on ExGap is significantly weakened, confirming that digital finance can alleviate the effect of population aging on inter-urban income gap.

## Further analysis

The previous research findings examine the impact of population aging on the average level of intra-urban income gap and inter-urban income gap. Nevertheless, in practice, we pay greater attention to how the marginal impact effect of population aging varies at different levels of intra-urban income gap and inter-urban income gap. Therefore, this study employs the analytical approach of the panel quantile regression model to investigate the evolutionary trajectory of the impact effects of population aging on intra-urban income gap and inter-urban income gap. Specifically, this paper constructs the panel quantile regression models as presented in Equations (10) and (11).


$$Q_{\tau }(InGap_{\;i,\;t}\;|\;{Agi}_{t})=\varphi_{\tau 0}+\varphi \tau 1InGapt+\eta_{\tau }Controls_{\;i,\;t}+\lambda i+\varepsilon_{\;i,\;t}$$ (10)

$$Q_{\tau }(\text{Ex }Gap_{\;i,\;t}\;|\;{Agi}_{t})=\varphi_{\tau 0}+\varphi_{\tau 1}\text{Ex}{Gap}_{\;t}+\eta_{\tau }Controls_{\;i,\;t}+\lambda_{i}+\varepsilon_{\;i,\;t}$$ (11)

Among them, Q_τ_(InGap_i,t_|Agi_t_) and Q_τ_(ExGap_i,t_|Agi_t_) respectively denote the values of intra-urban income gap and inter-urban income gap at the τ-th quantile given the population aging level Agi_t_. φ_τ1_ represents the regression coefficient of population aging at the τ-th quantile, φ_τ0_ denotes the intercept term at the τ-th quantile. This study selects five quantiles (0.10, 0.25, 0.50, 0.75, and 0.90) for analysis and elaboration.

Table 13 reports the regression results of the panel quantile model regarding the impact of population aging on intra-urban income gap. Columns (1) to (5) respectively present the regression outcomes at the 0.10, 0.25, 0.50, 0.75, and 0.90 quantiles of intra-urban income gap. It can be observed that as the quantile level increases, the marginal impact effect of population aging on intra-urban income gap generally demonstrates a trend of first rising and then declining. This phenomenon can be explained by the following mechanisms: at low and medium quantiles, the intra-urban income gap is relatively narrow, population aging initially widens the intra-urban income gap through labor supply contraction and industrial structure adjustment, resulting in a gradual strengthening of its impact effect. At high quantiles, the intra-urban income gap has already reached a high level and the social structure has become highly rigid. As a “systemic shock”, the marginal effect of population aging in “widening” the existing income gap naturally diminishes. Meanwhile, against the realistic institutional and social backdrop of China, when the intra-urban income gap becomes excessively large, the government will curb its further expansion through social welfare policies, taxation, and redistribution mechanisms, which in turn weakens the income gap widening effect induced by population aging.

**Table 13:** Population aging and the intra-urban income gap: Panel quantile model regression results.

	(1)	(2)	(3)	(4)	(5)
	τ = 0.10	τ = 0.25	τ = 0.50	τ = 0.75	τ = 0.90
Agi	0.979*** (0.215)	1.277*** (0.207)	1.328*** (0.119)	1.231*** (0.107)	1.218*** (0.112)
Invest	−0.648*** (0.131)	−0.773*** (0.084)	−0.548*** (0.071)	−0.644*** (0.051)	−0.602*** (0.058)
Finan	0.019*** (0.006)	0.005 (0.005)	−0.003 (0.005)	−0.006 (0.005)	−0.007 (0.005)
Health	−0.034 (0.027)	−0.017 (0.022)	−0.038 (0.026)	−0.047*** (0.012)	−0.042** (0.018)
Automa	0.026*** (0.009)	0.041*** (0.009)	0.043*** (0.007)	0.039*** (0.008)	0.030** (0.013)
Cons	0.308*** (0.073)	0.449*** (0.081)	0.482*** (0.084)	0.693*** (0.026)	0.691*** (0.064)
Fixed region	Yes	Yes	Yes	Yes	Yes
Fixed time	Yes	Yes	Yes	Yes	Yes
N	3408	3408	3408	3408	3408
R^2^	0.217	0.348	0.449	0.513	0.510

Table 14 presents the regression results of inter-urban income gap at the 0.10, 0.25, 0.50, 0.75, and 0.90 quantiles. It can be seen that as the quantile level of inter-urban income gap increases, the impact coefficient of population aging on inter-urban income gap generally decreases. This paper explains this phenomenon as follows: regions with a narrow inter-urban income gap typically enjoy robust economic development and higher income levels. Their sustained growth hinges on the continuous influx of high-quality human capital and the maintenance of a dynamic innovation ecosystem. The deepening of population aging directly erodes this foundation, and the high labor costs amplify the pension burden, resulting in a significant impact on inter-urban income gap. Conversely, regions marked by a wide inter-urban income gap generally experience lower economic development and income levels. Their labor markets are dominated by low-skilled competition, and income distribution remains in a state of low-level equilibrium. Under these conditions, the potential impact of population aging on income distribution is often overshadowed by more pressing developmental shortcomings, rendering its effect on the inter-urban income gap relatively limited.

**Table 14:** Population aging and the inter-urban income gap: Panel quantile model regression results.

	(1)	(2)	(3)	(4)	(5)
	τ = 0.10	τ = 0.25	τ = 0.50	τ = 0.75	τ = 0.90
Agi	1.995*** (0.434)	1.744*** (0.378)	1.760*** (0.313)	1.273*** (0.356)	0.913*** (0.255)
Invest	−1.504*** (0.193)	−1.520*** (0.208)	−1.475*** (0.169)	−0.890*** (0.260)	−1.060*** (0.199)
Finan	−0.006 (0.019)	−0.041*** (0.011)	−0.056*** (0.017)	−0.088*** (0.021)	−0.151*** (0.026)
Health	−0.833*** (0.063)	−0.671*** (0.055)	−0.576*** (0.047)	−0.509*** (0.055)	−0.271*** (0.097)
Automa	−0.062*** (0.021)	−0.082*** (0.014)	−0.103*** (0.011)	−0.124*** (0.023)	−0.106*** (0.038)
Cons	1.602*** (0.082)	1.618*** (0.073)	1.658*** (0.066)	1.716*** (0.089)	1.786*** (0.079)
Fixed region	Yes	Yes	Yes	Yes	Yes
Fixed time	Yes	Yes	Yes	Yes	Yes
N	3408	3408	3408	3408	3408
R^2^	0.445	0.427	0.423	0.433	0.441

## Conclusions and policy implications

Utilizing the panel data of 284 prefecture-level cities in China spanning from 2011 to 2022, this paper empirically examines the impact and mechanism of population aging on the urban income gap through regression analysis. Additionally, it explores the moderating effect of digital finance. The main conclusions are as follows: firstly, population aging exerts a significantly positive influence on both the intra-urban and the inter-urban income gap. After conducting a series of rigorous robustness tests, these results still hold strong significance. The heterogeneity analysis indicate that the impact of population aging on the intra-urban income gap does not exhibit significant differences across different city tiers and resource endowments. While the impact of population aging on the inter-urban income gap cities is more significant in the samples of underdeveloped cities and resource-based cities. Secondly, the results of mechanism analysis indicate that population aging can reduce the labor income share and increase social security expenditure, which in turn widen the intra-urban income gap. Meanwhile, population aging enlarges the inter-urban income gap by reducing the technological innovation and impeding industrial structure upgrading. Finally, digital finance effectively alleviates the impact of population aging on the intra-urban income gap and inter-urban income gap. Further research suggests that the impact of population aging on income gaps is not linear but exhibits distinct non-linear patterns. Specifically, as the quantile of the intra-urban income gap increases, the marginal impact effect of population aging on intra-urban income gap generally demonstrates a trend of first rising and then declining. Conversely, for the inter-urban income gap, the corresponding impact coefficient monotonically decreases as the quantile rises.

Based on the findings, the following policy recommendations are proposed.

Firstly, promote collaboration among the government, financial institutions, and communities to implement targeted digital literacy improvement plans and financial technology subsidy policies. Based on the community elderly care service center, we will build a “Silver Hair Digital Classroom”, develop age friendly teaching materials with illustrations and text, and combine dialect teaching videos with one-on-one practical guidance. We will focus on training basic skills such as mobile payment, pension inquiry, and fraud prevention. We will also collaborate with technology companies to develop a simplified financial app, and provide traffic subsidies and smart terminal trade in coupons to elderly people who participate in the training. Implement “aging friendly transformation subsidies” for financial institutions, provide tax reductions based on the activity level of elderly users and the evaluation results of product usability, encourage banks to reduce interbank transfer fees for elderly people, and lower online insurance thresholds for insurance companies. At the same time, set up special funds in central and western cities to provide monthly targeted free data and low-priced smartphone rental services for rural elderly groups. By reducing usage costs and skill barriers, digital finance can truly become a universal tool that transcends age and region.

Secondly, we will focus on building a digital financial support system that is adapted to an aging society, in order to narrow the income gap through inclusive development. Within the city, promote financial institutions to optimize aging friendly services, simplify operational processes and strengthen risk warnings, rely on community networks to promote financial knowledge, help elderly people overcome technological barriers, and ensure that different income groups can conveniently use basic financial services. Through policy guidance, promote the balanced layout of digital financial resources between cities, encourage financial institutions to extend services to areas with higher aging levels, improve the construction of grassroots digital service stations, and break the uneven distribution of resources caused by geographical limitations. At the same time, we will strengthen regulatory coordination, standardize the design of digital financial products, avoid excessive commercialization that may cause new exclusion to the elderly population, and use policy incentives and mechanism innovation to make digital finance an effective link to balance the income gap between urban and rural areas and regions.

Finally, establish a multidimensional policy support system. Within the city, the mechanism for increasing labor remuneration should be improved, a collective wage negotiation system led by trade unions should be established, and the share of labor income should be included in local government assessment indicators. At the same time, the structure of social security expenditures should be optimized, and pension subsidies should be implemented for low-income elderly groups. The property income of high-income groups should be regulated through a progressive tax system to avoid exacerbating the gap in social security expenditures due to differences in payment bases. Between cities, it is necessary to establish a special fund for entrepreneurship in aging cities, provide tax reductions and exemptions for new business models that absorb elderly labor, and establish a cross regional industrial coordination mechanism to support the development of the silver economy industry chain in cities with high aging levels through central government transfer payments. It is mandatory to require developed cities in the east and aging cities in the central and western regions to carry out industrial co construction, and link technological innovation and industrial upgrading speed with local government special debt quotas, forcing resources to flow to aging cities and curbing the widening gap from both income distribution sources and economic development momentum.

To gain a more profound understanding of the way digital finance influences the income gap induced by population aging, future research is needed.

On one hand, the present study is centered around the data analysis of China, and its findings cannot be effectively generalized to other countries. China has a distinct national context, with marked differences from other nations in aspects such as the political system, economic development trajectory, cultural heritage, and social structure. These multifaceted disparities render it arduous to directly apply the research conclusions derived from Chinese data to other countries, thus constraining the global dissemination and application of the research results. For future research, it is imperative to conduct in-depth cross-border comparative studies. Different representative countries and regions should be selected. We need to comprehensively take into account the variances in politics, economy, culture, society, and other dimensions among countries, and then analyze how these factors influence the interaction mechanisms among population aging, financial development, and the income gap.

On the other hand, in the research process, the research perspective mainly stays at the static level. When analyzing the impact of population aging on urban income gap, more attention is paid to the direct relationship between the two in a specific period, but the dynamic change process of population aging over time is not fully considered, such as the change of aging speed, the evolution of the elderly population structure, etc. Future research should shift from static analysis to dynamic research, and build a dynamic model to explore the dynamic relationship between population aging, digital finance and urban income gap. For example, by using dynamic panel model and other methods, considering the interaction and feedback mechanism of different variables at different time points, this paper analyzes how the urban income gap dynamically evolves with the gradual deepening of population aging and the continuous development of digital finance, and what policies and measures can be adopted at different stages to more effectively adjust the income gap.

## Supporting information

S1 File Supporting information 1. Dataset used in this study extracted from China Statistical Yearbook public profiles.
(XLSX)
